# FUNDOPLICATION CONVERSION IN ROUX-EN-Y GASTRIC BYPASS FOR CONTROL OF OBESITY AND GASTROESOPHAGEAL REFLUX: SYSTEMATIC REVIEW

**DOI:** 10.1590/0102-6720201700040012

**Published:** 2017

**Authors:** Antônio Moreira MENDES-FILHO, Eduardo Sávio Nascimento GODOY, Helga Cristina Almeida Wahnon ALHINHO, Manoel dos Passos GALVÃO-NETO, Almino Cardoso RAMOS, Álvaro Antônio Bandeira FERRAZ, Josemberg Marins CAMPOS

**Affiliations:** 1 Post-Graduation Program in Surgery, Federal University of Pernambuco, Recife, PE;; 2 Gastro Obeso Center Clinic, São Paulo, SP;; 3 Department of Surgery and Clinical Medicine, Federal University of Pernambuco, Recife, PE, Brazil

**Keywords:** Bariatric surgery, Gastroesophageal reflux, Gastric bypass, Fundoplication, Laparoscopy., Cirurgia bariátrica, Refluxo gastroesofágico, Derivação gástrica, Fundoplicatura, Laparoscopia

## Abstract

*****Introduction*** ****:**

Obesity is related with higher incidence of gastroesophageal reflux disease. Antireflux surgery has inadequate results when associated with obesity, due to migration and/or subsequent disruption of antireflux wrap. Gastric bypass, meanwhile, provides good control of gastroesophageal reflux.

***Objective*:**

To evaluate the technical difficulty in performing gastric bypass in patients previously submitted to antireflux surgery, and its effectiveness in controlling gastroesophageal reflux.

***Methods*:**

Literature review was conducted between July to October 2016 in Medline database, using the following search strategy: (“Gastric bypass” OR “Roux-en-Y”) AND (“Fundoplication” OR “Nissen ‘) AND (“Reoperation” OR “Reoperative” OR “Revisional” OR “Revision” OR “Complications”).

***Results*:**

Were initially classified 102 articles; from them at the end only six were selected by exclusion criteria. A total of 121 patients were included, 68 women. The mean preoperative body mass index was 37.17 kg/m² and age of 52.60 years. Laparoscopic Nissen fundoplication was the main prior antireflux surgery (70.58%). The most common findings on esophagogastroduodenoscopy were esophagitis (n=7) and Barrett’s esophagus (n=6); the most common early complication was gastric perforation (n=7), and most common late complication was stricture of gastrojejunostomy (n=9). Laparoscopic gastric bypass was performed in 99 patients, with an average time of 331 min. Most patients had complete remission of symptoms and efficient excess weight loss.

***Conclusion*:**

Although technically more difficult, with higher incidence of complications, gastric bypass is a safe and effective option for controlling gastroesophageal reflux in obese patients previously submitted to antireflux surgery, with the added benefit of excess weight loss.

## RESUMO

***Introdução*:**

Obesidade está relacionada à maior incidência de doença do refluxo gastroesofágico. Cirurgia antirrefluxo apresenta resultados inadequados quando associada à obesidade, devido à migração e/ou ruptura posterior da válvula antirrefluxo. Bypass gástrico enquanto isso determina bom controle de refluxo gastroesofágico.

***Objetivo*:**

Avaliar a dificuldade técnica na realização de bypass gástrico em pacientes previamente submetidos à cirurgia antirrefluxo, e sua eficácia no controle do refluxo gastroesofágico.

***Método*:**

Revisão de literatura foi realizada entre os meses de julho a outubro de 2016, na base de dados Medline, com a seguinte estratégia de busca: (“Gastric Bypass” OR “Roux-en-Y”) AND (“Fundoplication” OR “Nissen”) AND (“Reoperation” OR “Reoperative” OR “Revisional” OR “Revision” OR “Complications”).

Foram inicialmente classificados 102 artigos selecionando-se, por critérios de exclusão, apenas seis ao final. Foram incluídos 121 pacientes, sendo 68 mulheres. A média de índice de massa corporal pré-operatório foi 37,17 kg/m² e idade de 52,60 anos. Fundoplicatura de Nissen laparoscópica foi a principal operação antirrefluxo prévia (70,58%). Os achados mais comuns na endoscopia digestiva alta foram esofagite (n=7) e esôfago de Barrett (n=6); a complicação precoce mais comum foi perfuração gástrica (n=7), e tardia, estenose de anastomose gastrojejunal (n=9). Bypass laparoscópico foi realizado em 99 pacientes, com tempo médio de 331 min. A grande maioria dos pacientes apresentou completa remissão dos sintomas e perda eficiente do excesso de peso**.**

***Conclusão*:**

Apesar de tecnicamente mais difícil, com maior incidência de complicações, o bypass gástrico é opção segura e efetiva no controle do refluxo gastroesofágico em pacientes obesos previamente submetidos à operação antirrefluxo, com a vantagem adicional da perda do excesso de peso.

## INTRODUCTION

Gastroesophageal reflux disease (GERD) has a prevalence estimated between 20-40% in the USA and Europe and 12% in Brazil^6^ ^,^ ^14^ ^,^ ^15^ ^,^ ^22^. Obesity is a frequently associated condition, due to an increase in abdominal pressure with consequent hypotonia of the lower esophageal sphincter, and increase in the frequency of its spontaneous relaxation^1^ ^,^ ^3^ ^,^ ^5^ ^,^ ^10^ ^,^ ^19^ ^,^ ^20^. The surgical treatment of GERD has regained space with the advent of laparoscopy. However, the results in obese patients are deficient, and partial or total rupture and even migration of the anti-reflux valve may occur^13^. On the other hand, gastric bypass (GB), a surgery widely used to treat morbid obesity, has excellent results in the control of gastroesophageal reflux^8^. In recent years, it has become an alternative for recurrence of GERD after fundoplication, although it is associated with greater difficulties and complications^11^ ^,^ ^14^.

This systematic review aims to evaluate the efficacy and safety, analyzing the technical difficulties and complications of GB in the control of GERD, in patients previously submitted to antireflux surgery.

## METHODS

### Search strategy

A systematic review of the literature was performed from July to August 2016 in the Medline database, using the following cross-referencing of Boolean terms and headings: (“Gastric bypass” OR “Roux-en-Y”) AND “Nissen”) AND (“Reoperation” OR “Reoperative” OR “Revision” OR “Revision” OR “Complications”).

### Articles selection

#### *Inclusion criteria*

Full original articles were searched, published in English, from 1995 to 2016, in which GB was used to treat GERD recurrence after antireflux operation

#### *Exclusion criteria*

Case reports (or series), review articles and articles with the use other surgical techniques were excluded.

#### *Evaluated variables*

The number of patients operated, operative time, hospitalization time and reported complications were the extracted data (Table 1).

**TABLE 1:** Data extracted from each study

Autor	n	Fundoplicatura prévia	Achados EGD	Válvula à EGD	Tipo Bypass	IMC pré- operatório (Kg/m’)	Tempo de Operação (minutos)	Tempo de Internamento (dias)	IMC pós- operatório (Kg/m!)	Remissão dos sintomas	Uso de Medicação Anti-refluxo
Raftopoulos l et al. 2004	7	4 LNF 2LNCF 1 ONF	1 refluxo 1 gastrite 1 obstrução da JGE 1 estenosedeJGE	4 intactas 3 hérnias hiatais (1 deslizamento torácico)	7LGBP	37,5	372(206-523)	4,8 (3-8)	26,8	Total: 1/7 Parcial: 6/7	Nenhuma: 3/7 IBP: 3 BH2:1
Houghton SG et al. 2005	19	10 LNF 7 ONF 1 Nissen transtoracica 1 Toupet	3 esofagite 2 Barrett 1 erosão Cameron	9 intactas 4 hérnias hiatais recorrentes 1 “slipped Nissen”	170GB 2LGB	42	NR	7	32 +-2	Total: 18/18	Nenhuma:18/18
Kellogg TA et al. 2007	11	8 LNF 3 ONF	4 esofagite erosiva	NR	11 LGB	44	349(222-624)	3,4 (2-6)	30,2	Total:7/9 Parcial:2/9	NR
IbeleAetal. 2012	14	14 LNF	NR	NR	14LGBP	43,5	160(120-240)	5,1 (1-17)	NR	Total: 14/14	Nenhuma: 8/10 IBP 2/10
Stefanidis D et al. 2012	25	NR	NR	14 hérnias hiatais 7 rupturas	24 LGB 10GB	34,4	345(180-600)	7(2-30)	60% do sobrepeso	Total: 24/25 Parcial: 1/25	NR
Kim Metal. 2014	45	NR	4 Esôfago de Barrett	9 rupturas 25 hérnias hiatais	41 LGB 4 0GB	33	367(190-600)	4(1-33)	52.6% do sobrepeso	Total: 42/45 Parcial: 3/45	NR

### Selected papers

**FIGURE 1:**
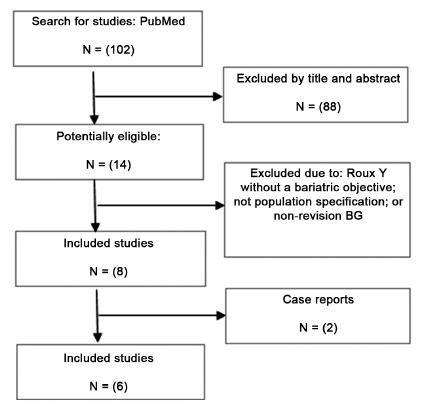
Systematic review flowchart

One hundred and two articles were found with the search strategy; 88 were initially excluded by title and abstract. Of the remaining 14, eight were excluded after reading the full text, as they did not meet the inclusion criteria, resulting in a final number of six articles^11^ ^,^ ^12^ ^,^ ^13^ ^,^ ^14^ ^,^ ^17^ ^,^ ^22^. Figure 1 illustrates the research strategy

## RESULTS

### Characteristics of the studies

Six were selected for analysis, comprising 121 patients. Published papers were from the USA. Publication date ranged from 2004 to 2014.

### Characteristics of the patients (n=121)

Four of the six studies reported the first surgical technique: 36 laparoscopic Nissen fundoplications (70.58%); 11 laparotomic Nissen fundoplications (21.56%); two laparoscopic Nissen-Collis fundoplication (3.92%); one transthoracic Nissen fundoplication (1.96%); and one Toupet fundoplication (1.96%). In the rest (n=70) no information about performed technique was found. The mean preoperative body mass index (BMI) was 37.17 kg/m² (21.6-50.6 kg/m²), with a mean age of 52.60 years (25-74). Five studies reported gender of the patients: 68 patients were women (89.47%) and eight men (10.53%). Table 2 presents the anthropometric data.

**TABLE 2:** Anthropometric data

Number of patients	121
BMI - mean (kg/m²)	37.17 (21.6 - 50.6)
Age - average (years)	52.60 (25 - 74)
Gender (reported in five studies)	89.47% F / 10.53% M

### Preoperative upper gastrointestinal endoscopy findings

It was performed and reported in 96 patients; all, except one study, reported the status of the fundoplication valve: seven had intact wrap; 21 with ruptures; one slip; one partial herniation; one herniation with slip; and one distorted. There were also 46 hiatal hernias.

The most frequent endoscopic alterations were esophagitis (n=7) and Barrett’s esophagus (n=6). Reflux, gastritis, gastroesophageal junction obstruction, gastroesophageal junction stenosis and Cameron lesions were also found, with one case each.

### Revisional operation

All studies reported the revisional operation approach. The majority was submitted to laparoscopic GB (n=99) and 22 to laparotomic GB (81.81% vs. 18.18%). The mean surgical time was 331 min (180-624) and the mean length of hospital stay was 5.21 days (1-33).

### Complications

They were classified as precocious (≤30 days) or late (> 30 days) in four studies.

The most common precocious was gastric perforation (n=7), followed by intestinal obstruction (n=4), operative wound infection (n=4), fistula in gastrojejunal anastomosis (n=3), hemorrhage (n=2), pulmonary embolism (n=2), splenectomy (n=1), pressure ulcer (n=1) and pneumonia (n=1, Table 3).

**TABLE 3:** Early complications

Complication	n (%)
Gastric perforation	7 (5.78)
Bowel obstruction	4 (3.30)
Surgical wound infection	4 (3.30)
Leakage of gastrojejunal anastomosis	3 (2.48)
Bleeding	3 (2.48)
Esophageal perforation	2 (1.65)
Pulmonary embolism	2 (1.65)
Splenectomy	1 (0.83)
Pressure ulcer	1 (0.83)
Pneumonia	1 (0.83)

The treatment of gastric perforation was detailed: in six cases was located at the fundus and resected at the gastrectomy; in one was repaired with suture, without sequelae. In relation to the two esophageal perforations, one was treated with gastric fundus patch; in the other no treatment details were mentioned^2^ ^,^ ^3^.

The most common late complications were gastrojejunal anastomotic stenosis (n=9), gastrojejunal fistulae (n=2), intestinal obstruction (n=4), gastrocutaneous fistula (n=2), marginal ulcer (n=2), gastrojejunal obstruction (n=2), gastrojejunal bleeding (n=2), perforation of duodenal diverticulum (n=2), respiratory failure (n=2), gastric herniation (n=1), internal hernia (n=1), pneumonia (n=1), nausea (n=1), vomiting (n=1), melena (n=1) and prolonged mechanical ventilation (n=1, Table 4).

**TABLE 4:** Late complications

Complication	n (%)
Gastrojejunal anastomosis stenosis	9 (7.44)
Bowel obstruction	4 (3.31)
Leakage of gastrojejunal anastomosis	2 (1.65)
Gastrocutaneous fistula	2 (1.65)
Marginal ulcer	2 (1.65)
Gastrojejunal obstruction	2 (1.65)
Gastrojejunal bleeding	2 (1.65)
Perforation of duodenal diverticulum	2 (1.65)
Respiratory insufficiency	2 (1.65)
Gastric herniation	1 (0.83)
Internal hernia	1 (0.83)
Cholecystitis	1 (0.83)
Pneumonia	1 (0.83)
Nausea	1 (0.83)
Vomiting	1 (0.83)
Melena	1 (0.83)
Prolonged mechanical ventilation	1 (0.83)

The treatment of gastrojejunal stenosis was reported in all cases. Balloon dilatation was the endoscopic procedure of choice, with success in all patients; in one case there was also a need for gastrostomy feeding through the stomach excluded because of concomitant gastrocutaneous fistula, which was resolved with the treatment^11^. The number of dilatations was reported in six patients, with an average of 3.5 (range of 1-6 sessions). The two patients who presented fistula in the gastrojejunal anastomosis were reoperated, but the techniques were not detailed^14^ ^,^ ^22^.

### Efficacy in controlling GERD

Regarding the efficacy of GB in the management of GERD, among the 118 patients who remained on follow-up, 106 presented total remission of GERD symptoms (89.8%), while the remaining 12 showed partial improvement (10.2%)^11^ ^,^ ^12^ ^,^ ^13^ ^,^ ^14^ ^,^ ^17^ ^,^ ^22^. Three studies reported maintenance of antireflux medications: of the 35 patients who used these drugs in the preoperative period, 29 no longer used (82.9%) and six maintained the use (17.1%)^11^ ^,^ ^12^ ^,^ ^17^.

## DISCUSSION

Many authors have already reported the poor outcome of the antireflux operation in obese patients, with valve migration or rupture in most cases^13^ ^,^ ^16^ ^,^ ^22^ ^,^ ^24^; others demonstrated different results, with similar efficacy to those performed on normal weight subjects. However, the latter have limitations in relation to the number of patients, follow-up period and the fact that most individuals were carriers of mild obesity^7^ ^,^ ^24^. GB has become the treatment of choice for GERD in this situation; its good results come from the fact that the small pouch contains few acid producing parietal cells and that the long alimentary loop (usually 1 m) prevents the return of biliopancreatic content^2^.

Bariatric revision procedures are more complex^18^ ^,^ ^26^, with fundoplication for GB being the group of higher risk when compared to the gastric band for vertical gastrectomy and gastric band for GB^21^. This in patients with anterior fundoplication has more technical difficulties, longer operative time and postoperative morbidity, both early and late period^9^ ^,^ ^21^. The technical difficulty of the revisional GB was well reported in the case-control study of Ibele et al.^12^. The revision was compared to GB without prior antireflux operation, with higher complication rates in the first group.

The technical difficulties usually reported are due to the occurrence of strong adhesions between liver and stomach, as well as the need to undo the anterior fundoplication region, to avoid making gastric pouch septa. This stage is responsible for the most common early postoperative complication: gastric perforation (n=7). All cases were treated by gastric fundus resection during GB, except one, in which the perforation was sutured^14^ ^,^ ^22^.

Gastrojejunal anastomosis stenosis was the late complication most reported in this review (n=9). It was more frequent in Raftopoulos et al report, occurring in five of the seven patients; these authors justified the fact due to the inclusion of patients already submitted to dilations in previous procedures (fundoplications and fundoplication redo)^17^. However, all were successfully treated with endoscopic dilation^11^ ^,^ ^12^ ^,^ ^17^.

Ibele et al. sugested maintaining fundoplication intact as an alternative to decrease the incidence of complications; however, the authors themselves question the option of not allowing adequate control of GERD or determining unsatisfactory weight loss^12^.

In a retrospective study, Kim et al. presented the initial results of the robotic technique (n=13), reporting a better intraoperative visualization as a possible advantage over traditional laparoscopic surgery, facilitating the dissection of hyaline adhesions and anterior fundoplication release; new studies are needed with this technique to assess whether there will be an impact on the reduction of complications^14^.

Laparoscopic approach was performed in 17 patients in the Houghton et al. series; however, it was associated with an extended hospital stay (seven days) and a complication rate of approximately 21%^11^.

The redo fundoplication technique, indicated by some authors as an alternative to the initial fundoplication failure^23^ shows inadequate results, with failure rates above 60% in 10 years^4^. Kim et al. reported GB patients who had undergone three previous fundoplications.

Weight gain after initial antireflux operation was identified as the main cause of failure and reported in all included articles; the majority of patients undergoing the new procedure had grade I obesity (some grade II); all authors pointed to the efficient excess weight loss after GB as an additional advantage^12^ ^,^ ^13^ ^,^ ^14^ ^,^ ^17^ ^,^ ^22^ ^,^ ^25^.

## CONCLUSION

Despite a higher rate of postoperative complications, GB is a safe and effective option to control GERD after the failure of the antireflux operation in obese patients, with the additional advantage of losing excess weight.
